# The Microstructure Evolution of a Fe_3_Al Alloy during the LENS Process

**DOI:** 10.3390/ma11030390

**Published:** 2018-03-07

**Authors:** Krzysztof Karczewski, Tomasz Durejko, Tomasz Czujko

**Affiliations:** Department of Advanced Materials and Technologies, Military University of Technology, ul. Gen. Urbanowicza 2, 00-908 Warsaw, Poland; krzysztof.karczewski@wat.edu.pl (K.K.); tomasz.durejko@wat.edu.pl (T.D.)

**Keywords:** LENS, intermetallic alloys, microstructure

## Abstract

A Fe_3_Al intermetallic alloy has been successfully prepared by the laser-engineered net shaping (LENS) process. The applied process parameters were selected to provide various cooling rates during the solidification of the laser-melted material. The macro- and microstructure and the micro- and macrotexture of Fe_3_Al samples were investigated. The influence of the cooling rate on grain morphology and texture is discussed. For the applied cooling rate range of 0.64 × 10^4^ K/s–2.6 × 10^4^ K/s, the structure is characterized by the presence of columnar grains for which the growth is directed upwards from the substrate. The intensity of the microtexture varies with the height of the sample and the cooling rate. The intensity of the texture increases with the decrease in the cooling rate. The samples that were obtained with low and medium cooling rates are characterized by the well-developed <100> and <111> macrotextures. The Fe_3_Al alloy that was produced with a high cooling rate did not show a specific texture, which is reflected in the fairly uniform distribution of the normalized density intensity. Only a very weak texture with a <100> type component was observed.

## 1. Introduction

Ordered intermetallic alloys based on aluminides, especially FeAl (B2) and Fe_3_Al (D03) intermetallic phases, are known as materials with unique physical and mechanical properties, especially at elevated temperatures, as well as a high resistance to oxidation, corrosion wear [1,2,3,4,5,6,7], and creep above 600 °C [7,8]. Unfortunately, despite the extensive research and dynamic development of the technology sector, the production and processing of this type of alloys still exhibit many problems [9,10,11]. Therefore, the sintering or hot extrusion of elementary powders mixture was applied for producing the FeAl or Fe_3_Al alloys [4,10,11]. However, the application of powder technology to the manufacturing of FeAl- or Fe_3_Al-based components is associated with high energy consumption and low productivity, which lead to high production costs [12].

Rapid laser manufacturing technologies are currently attracting widespread interest in the fields of manufacturing parts made of metals and alloys. Direct deposition methods, especially laser-engineered net shaping (LENS) [13] are of particular significance in this area. LENS is a relatively new direct deposition additive manufacturing method that allows the direct production of metal components using a combination of the metal powders melting process with predesigned three-dimensional CAD models. In the LENS method, the designed part is fabricated “layer by layer” through the melting of metal powders supplied by powder feeders located around the high energy laser head. To date, the additive manufacturing (AM) of intermetallic-based alloys was only reported in a few papers [14,15,16]. Recently, we reported a successful fabrication of the Fe_3_Al/SS316L graded structure [17] and Fe_3_Al-0.35Zr-0.1B alloy [18], as obtained by using the laser-engineered net shaping (LENS) technique. However, dynamically changing process conditions, such as the substrate and preceding layer temperature, have a significant influence on the microstructure development of the subsequent layers. The morphology of the resulting grains strongly depends on the cooling rate in the crystallization zone. Depending on the cooling rate and the direction of heat dissipation, the formed structure can consist of frozen, columnar, or dendrite grains [18,19,20,21]. A highly textured structure gives rise to the anisotropy of thermal and mechanical properties of the fabricated elements.

The purpose of this study is to describe and examine the microstructure evolution and texture in the Fe_3_Al alloy during the LENS process with variable parameters providing the varied gradient of temperature in the melted metal pool, i.e., a different cooling rate.

## 2. Materials and Methods

**Figure 1:**
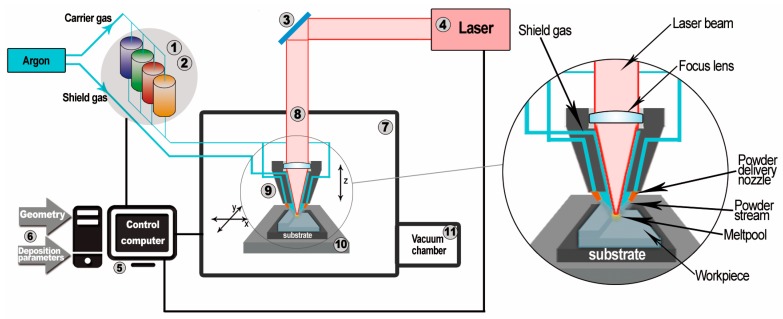
Scheme of the laser-engineered net shaping (LENS) system [17]: 1. Powder supply—it is possible to install and use a four powder supply unit; 2. Pneumatic vibrating system that provides stable powder doses; 3. Optical system for focusing the laser beam, the optical path, and the imager thermal system for analyzing the temperature distribution of the liquid metal; 4. IPG fiber laser with the wavelength of 1070 nm and power of 500 W; 5. Computers for process control; 6. Input data entered by the operator; 7. Working chamber with protective atmosphere with controlled oxygen and water vapor content (less than 10 ppm). Work in the chamber is conducted using butyl gloves and sight glass in the form of transparent plastic with a filter to protect the operator from high-power laser radiation; 8. Optical path of the laser; 9. Working head with four nozzles controlling the flow of powder into the laser beam focus zone; 10. Numerically controlled working table (movement in the *X*-*Y* plane); 11. Antechamber with atmosphere purification vacuum system.

The schematic diagram and working principles of the LENS MR-7 system that are applied in the present work are shown in Figure 1 [17]. The device is based on melting of the material supplied in the form of metal or intermetallic powders and using selective deposition on the substrate or on a previously built layer using a 0.5 kW continuous wave IPG YLR-500 fiber laser (OPTOMEC, Albuquerque, NM, USA). Highly pure, chemically homogeneous alloyed metal powders with good metallurgical quality (e.g., a lack of porosity), a spherical shape, and a granulation of 44–150 μm are usually used as an initial batch in the LENS technique [13]. The application of high-quality powders ensures the good quality of the obtained final products. This technique allows for the simultaneous forming of a microstructure and geometry of the manufactured component through precise control of working parameters such as the powder flow rate, the laser power, the feed of the working table, and the heat transfer rate. The proper selection of process parameters has a crucial impact on the temperature gradient and the cooling rate, which, in turn, determine the microstructure of the processed samples.

**Figure 2:**
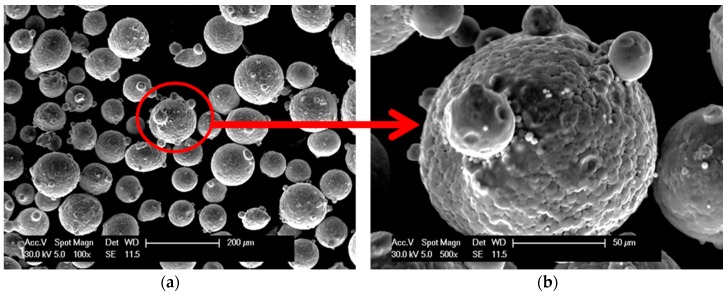
The morphology of Fe_3_Al powder particles at low (**a**) and high magnification (**b**).

The experimental material for LENS process was Fe_3_Al (Fe‒28 at % Al) intermetallic spherical powder (Figure 2). The Fe_3_Al powder particles used during the experiment were of a smaller size (25–90 μm) than that recommended by the LENS manufacturer due to their low porosity (approximately 0.1%) when compared to the identical stock with the particle size in the 40–150 μm range (approximately 0.3% porosity). The powder was deposited on substrates made of Fe Armco.

To study the microstructure evolution in Fe_3_Al alloys that were fabricated by the LENS technique, rectangular samples with dimensions of 10 mm × 10 mm × 5 mm were manufactured. The applied process parameters were selected to provide various cooling rates during the solidification of the laser-melted material. The cooling rate *dT*/*dt* was calculated from the formula:

(1) $$\frac{dT}{\;dt}=v\frac{dT}{dx}\;$$

where *v* is the head feed rate and *dT*/*dx* is the temperature gradient in the melted metal pool. The temperature gradient was determined using the thermal imager and ThermaViz 2.0 software. The thermal images were obtained at different heights of the constructed elements for the zone at the ground, half of the height, and the top of the sample. The applied processing parameters and obtained cooling rates are listed in Table 1.

Macrostructure observations were carried out with a Nikon Eclipse MA200 light microscope (Nikon Corporation, Tokyo, Japan). Prior to structural examinations, all of the samples were subjected to the following metallographic preparation procedure: grinding on SiC papers with the granulation in the sequence of 120, 240, 500, 1200, and 2400; polishing with diamond suspensions (3, 1, and 0.25 μm); and, finally polishing with silica suspensions of 0.1 and 0.06 μm. To reveal the microstructure, the samples were etched with Kalling’s reagent.

Microstructure evaluation was carried out using an FEI Quanta 3D field emission gun scanning electron microscope (FEG-SEM, Thermo Fisher Scientific, Hillsboro, OR, USA) that was equipped with an electron backscatter diffraction (EBSD) system. The EBSD technique was applied for insightful qualitative and quantitative microstructure measurements to obtain the grain size and shape, grain boundaries misorientation and for grains orientation analysis. All of the misorientation angles between the grains that were greater than 15° were considered to be high-angle boundaries. Microscopic observations were conducted on the longitudinal sections of the samples (across the wall thickness). Prior to structural examinations, all of the samples were subjected to the following metallographic preparation: grinding on SiC papers with a granulation up to 1200, polishing with diamond suspensions (3, 1, and 0.25 μm), and finally polishing with silica suspensions.

The macrotexture study was carried out using a Rigaku ULTIMA IV X-ray diffractometer (Rigaku Corporation, Tokyo, Japan) with a cobalt lamp equipped with the MPA-ML4 adapter (Rigaku Corporation, Tokyo, Japan). The macrotexture studies were carried out on the same samples that were selected for the microtexture studies in order to correlate the obtained micro- and macrotexture measurements. As in the case of the microtexture analysis, a reference system was defined, namely, the direction of growth was marked with BD, and the transverse direction was marked with TD. The measurement was made in the angular range α = 25–90° and β = 0–360° using the angular step of 5° for both angles. Pole figures for (220), (400), and (422) surfaces or equivalent planes were obtained. The obtained polar figures were normalized using TSL OIM Analysis 5.31 software.

## 3. Results and Discussion

### 3.1. The Macrostructure of Fabricated Samples

**Figure 3:**
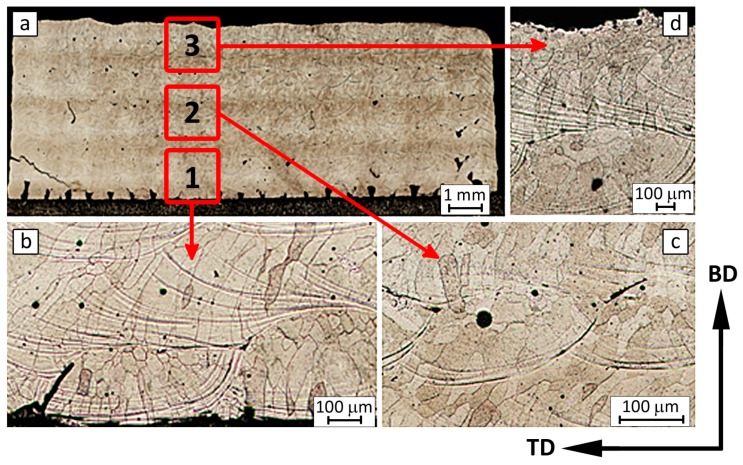
The macro (**a**) and microstructure of the Fe_3_Al alloy manufactured by LENS technique obtained at a high cooling rate at the substrate = bottom (**b**), half the height of the sample = middle, center (**c**), and top of the sample = surface (**d**).

The typical macrostructure of the Fe_3_Al alloy obtained by the LENS technique does not depend on the cooling rate and is presented in Figure 3. The macrostructure is characterized by the presence of columnar grains for which the growth is directed up from the substrate. These grains occur practically in the entire cross-section of the sample. However, the observed grains differ in size and shape. In addition, finer columnar grains can be observed with the substrate, while those that are in the center and top zones are larger. This variation probably results from the faster heat dissipation at the substrate than in the middle and upper parts of the sample. In turn, the shape of these grains is determined by the building strategy of successive layers, where each subsequent layer was built perpendicular to the previous one. The oriented columnar grains arise from the directional process of crystallization due to directional heat dissipation (columnar grains are parallel to the direction of heat dissipation). Furthermore, practically no frozen or equiaxed grains were observed, which are typically observed after AM [17,18]. However, it has been proved that only doping of Fe_3_Al alloys by Zr, B, or Nb, B can provide to equiaxed grain structure in the middle/top zones and all volume of the sample, respectively [18,19].

Such a highly textured structure characterized by the presence of large columnar grains spreading over several layers along the building direction has been widely observed in many materials fabricated by different additive methods [21,22,23,24,25,26]. Additionally, fine equiaxed grains that are near the substrate or previous layer surface have also been frequently observed [21,26]. The mechanism of columnar and fine equiaxed grain formation during the AM process was clearly described by Yan et al. [21]. The direct solidification on the substrate or re-melting of the previous layer during AM generally induces heterogeneous nucleation at the melt pool boundary and epitaxial grain growth with a cellular or dendritic solidification front. If the epitaxial growth of columnar grains is restrained by the formation of equiaxed grains near the surface of the melt pool, and the equiaxed grain depth within the melt pool is greater than the penetration depth during re-melting, the equiaxed grain size then dominates the average transverse grain size [21].

### 3.2. The Microstructure of Fe_3_Al Samples Fabricated by LENS

Because the macrostructure of the Fe_3_Al alloy produced by the LENS technique is not sensitive to the cooling rate, an in-depth analysis of the microstructure of this alloy was performed. The microstructure evaluation was carried out using the electron backscatter diffraction (EBSD) technique. The EBSD technique was applied for insightful qualitative and quantitative microstructure measurements of the grain size and shape, grain boundary misorientation, and grain orientation analysis.

**Figure 4:**
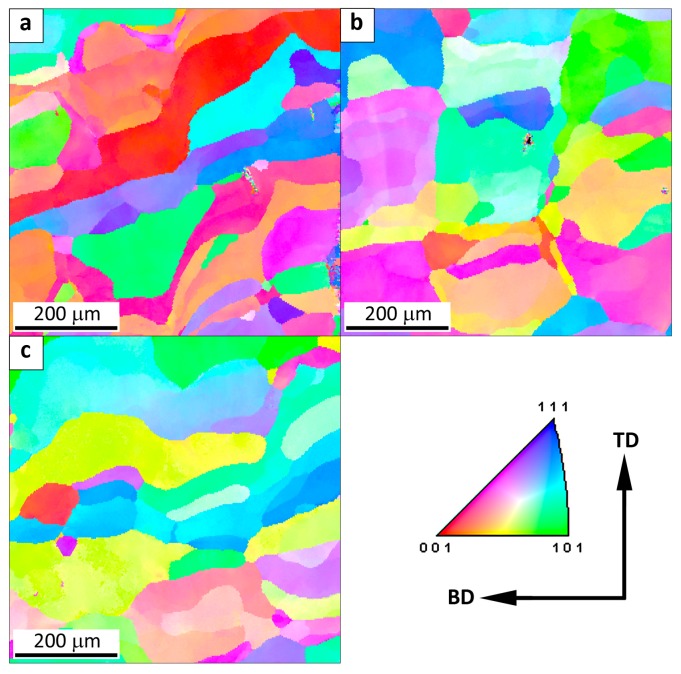
The map of crystallographic orientation in a sample obtained at a low cooling rate: (**a**) at the substrate (0.7 × 10^4^ K/s), (**b**) half the height of the sample (0.6 × 10^4^ K/s) and (**c**) top of the sample (0.6 × 10^4^ K/s). The spatial orientation of the sample is presented with respect to the building direction (BD) and transverse direction (TD).

The microstructure of the Fe_3_Al alloy obtained by the LENS technique at a low cooling rate (0.64 × 10^4^ K/s) is presented in Figure 4. The microstructure is characterized by the presence of elongated columnar grains parallel to the building direction (BD).

The direction of grain growth is related to the direction of heat transfer from the melt pool to the substrate. Due to the comparable cooling rate of 0.6 × 10^4^ K/s, both the shape and size of the grains are comparable at the substrate, half the height and the top of the sample. The average equivalent diameter of the grains ECD is equal to 72, 57, and 66 μm for bottom, middle, and top of the sample, respectively. The fractions of low- and high-angle boundaries in the entire volume of the sample are 44% and 56%, respectively. The high fraction of low-angle boundaries and the related presence of substructure are typical for Fe_3_Al alloys [1,6,9]. Columnar grains have been widely observed in the solidified region of the components made of many alloys, e.g., titanium-based, iron-based, nickel-based, and intermetallic alloys [18,20,22,23,25,26].

**Figure 5:**
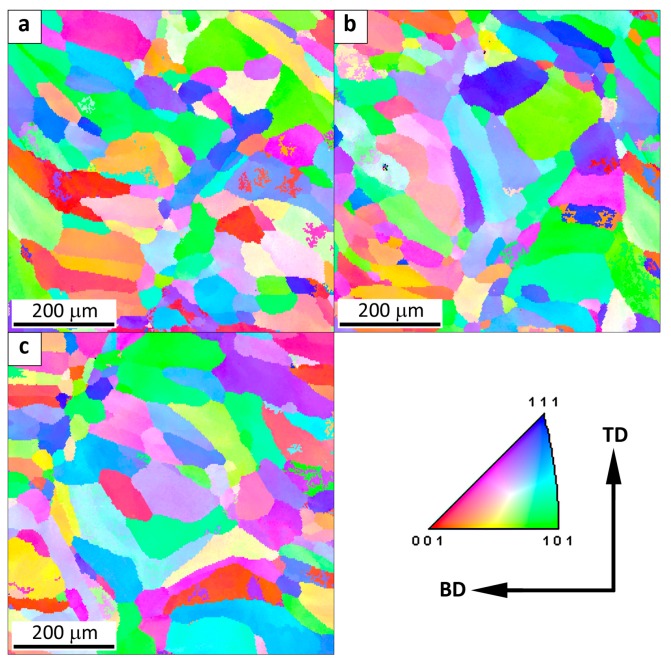
The map of crystallographic orientation in a sample obtained at a medium cooling rate: (**a**) at the substrate (2.1 × 10^4^ K/s), (**b**) half the height of the sample (1.6 × 10^4^ K/s) and (**c**) top of the sample (1.6 × 10^4^ K/s). The spatial orientation of the sample is presented with respect to the building direction (BD) and transverse direction (TD).

The microstructure of the Fe_3_Al alloy obtained by the LENS technique at a medium cooling rate is presented in Figure 5. As in the case of the samples that were manufactured at the low cooling rate, the microstructure is characterized by the presence of elongated columnar grains parallel to the building direction (BD). However, in addition to the columnar grains, fine equiaxed grains that are arranged along the boundary between successively deposited layers are also present. The presence of smaller equiaxed grains affects the value of the average equivalent diameter ECD = (4A/π)1/2 (A represents the projected grain area), which is equal to 44, 37, and 40 μm for the bottom, middle, and top of the sample, respectively. Regardless of the difference in the cooling rate at different heights (2.1 × 10^4^ K/s at the bottom and 1.6 × 10^4^ K/s in the middle and on the top of sample), the grain size is comparable. The fraction of low-angle grain boundaries is approximately 12%, which is much lower than for the samples that were fabricated at the low cooling rate.

**Figure 6:**
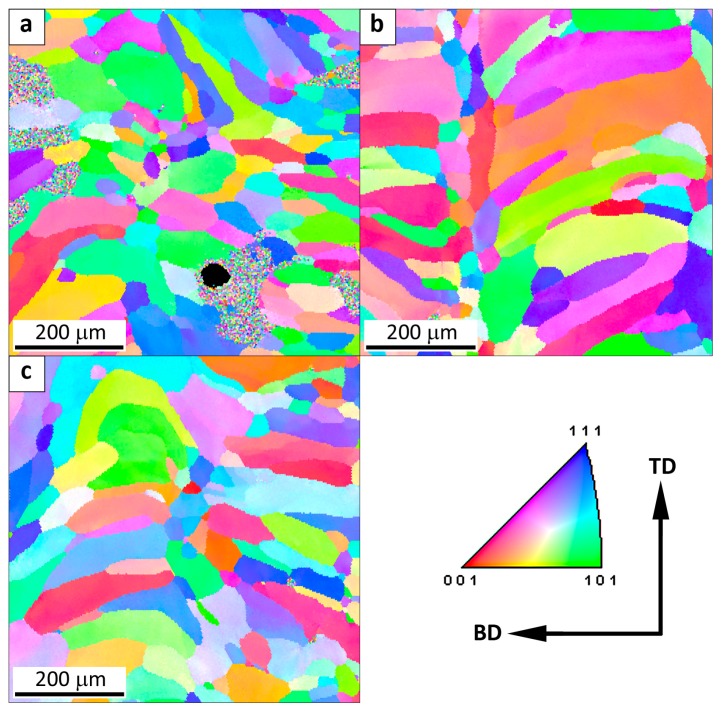
The map of crystallographic orientation in a sample obtained at a high cooling rate: (**a**) at the substrate (2.3 × 10^4^ K/s), (**b**) half the height of the sample (3.5 × 10^4^ K/s), and (**c**) top of the sample (2.6 × 10^4^ K/s). The spatial orientation of the sample is presented with respect to the building direction (BD) and transverse direction (TD).

On the other hand, the samples that were manufactured at a high cooling rate are characterized by the presence of both columnar and equiaxed grains (Figure 6). Due to the fact that the epitaxial growth of columnar grains can be restrained by the formation of equiaxed grains near the surface of the melt pool (next layer), and the equiaxed grain depth within the melt pool is greater than the penetration depth during re-melting, equiaxed grain size dominates the average transverse grain size. For AM processes, the equiaxed grain size is strongly determined by the number density of heterogeneous nucleation sites, which is usually easily controlled during the AM process [26,27]. The average grain size ECD is 40, 59, and 42 μm for the bottom, middle, and top of the sample, respectively.

It is noticeable that the increase in the cooling rate from 2.3 and 2.6 × 10^4^ K/s for the samples that were studied at the substrate and on the top (Figure 6a,c) to 3.5 × 10^4^ K/s in the middle of sample (Figure 6b) results in an increase in the fraction of columnar grains. The fraction of low-angle boundaries remains the same as that for the samples produced at the medium cooling rate and is equal to 12%. To summarize, the increase in the cooling rate from 0.6 to 3.5 × 10^4^ K/s results in the decrease in the columnar grain size and the decrease in the fraction of low-angle boundaries.

Moreover, the fine equiaxed grains arranged along the boundary between the successively deposited layers have been observed after the cooling rate increased to 1.6 × 10^4^ K/s.

### 3.3. The Micro- and Macrotexture of Fe_3_Al Samples Fabricated by LENS

#### 3.3.1. The Microtexture Results Obtained Using EBSD

**Figure 7:**
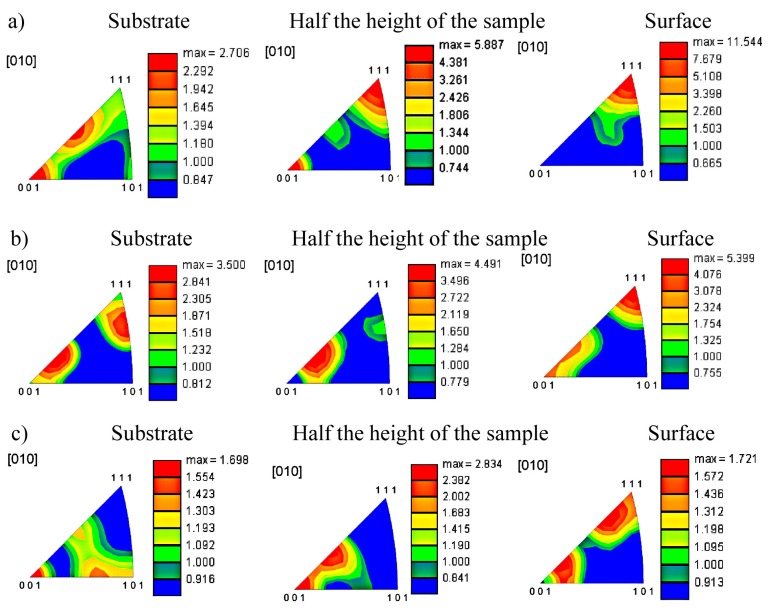
Inverse pole figures for samples made at (**a**) low (0.7, 0.6 and 0.6 × 10^4^ K/s), (**b**) medium (2.1, 1.6 and 1.6 × 10^4^ K/s), and (**c**) high (2.3, 3.5 and 2.6 × 10^4^ K/s) cooling rates.

Since texture strongly affects the mechanical properties of the additively manufactured components, the control of the texture based on scientific principles is important for the serviceability of the fabricated parts [28]. Since the morphology and micro- and macrotexture may differ for some alloys, first, an in-depth microtexture analysis was carried out. The inverse pole figures obtained for the samples produced at different cooling rates are shown in Figure 7. For the sample that was obtained at a low cooling rate (Figure 7a), a strong texture gradient on the sample thickness is observed. The least-developed texture is observed at the substrate, where a five-fold decrease in the intensity was found in relation to the texture at the surface of the sample. At the substrate, the texture is dispersed between the <111> and <100> poles, as well as <111> and <110> poles. In the middle part of the sample, a well-developed <100> and <111> texture is observed. On the surface of the sample, there is a strong texture (compared to the other analyzed samples) focused around the <111> direction.

For the sample obtained at a medium cooling rate (Figure 7b), the texturing intensity increases from the substrate to the top of the sample and a clear texture gradient with the thickness of the sample is observed. A well-formed texture dispersed between the <115> and <111> is observed at the substrate and in the middle of the sample. In the upper part of the sample, a highly developed fuzzy texture around <115> and <111> is found.

A quite different situation is observed for the sample manufactured with a high cooling rate. A very weak texture characterized by a dispersed, almost random grain orientation is observed near the substrate. A more developed texture in comparison to the texture observed near the substrate was found in the middle of the sample. The texture is focused around <115> and <100> poles. In the upper part, the texture intensity is similar to that of the middle part. However, the texture is focused around the <112> and <117> directions, which means a strong dispersion around <100>.

In summary, it should be stated that regardless of the changes in the cooling rate, the <100> texture is dominant in the Fe_3_Al alloy produced by the LENS technique. However, the level of dispersion from the dominant <100> texture increases noticeably with an increase in the cooling rate. Moreover, a very weak texture that is characterized by a dispersed, almost random grain orientation is observed near the substrate for the sample fabricated with the highest cooling rate of 3.5 × 10^4^ K/s. Finally, a strong texture gradient on the sample thickness is observed for the samples obtained at low and medium cooling rates (Figure 7a,b). The texture gradient is practically negligible for the Fe_3_Al alloy manufactured with a high cooling rate (Figure 7c).

#### 3.3.2. The Macrotexture Results Obtained by the Schultz Reflexive X-Ray Method

**Figure 8:**
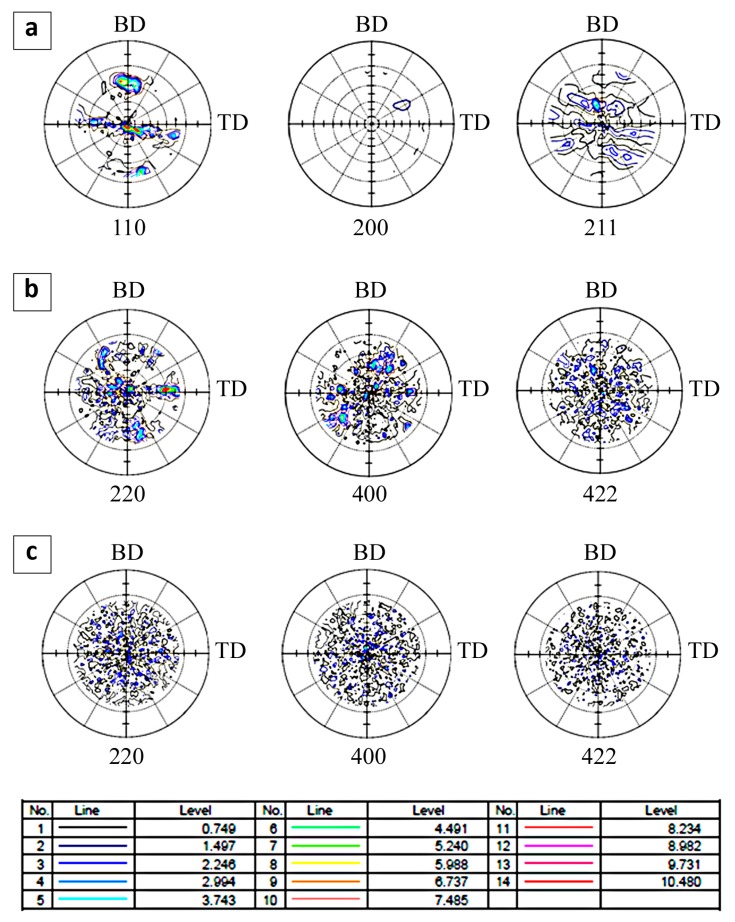
Incomplete pole figures and the legend of normalized intensity for the samples made at (**a**) low (0.6 × 10^4^ K/s), (**b**) medium (1.8 × 10^4^ K/s), and (**c**) high cooling rates (2.8 × 10^4^ K/s).

Incomplete polar figures for the Fe_3_Al phase with cubic crystal structure were obtained from the macrotexture analysis (Figure 8). The obtained results allow analysis of the texture development dependence on the temperature gradient accompanying the process of sample production. For the sample obtained at a low cooling rate (Figure 6a), a strong texture is observed.

The main texture components observed in the pole figures are <110> and <111>, which are inclined to the substrate at the angle of ~45°. In the remaining pole figures, i.e., (200) and (211), no clearly visible texture is observed. For the sample that was obtained at a medium cooling rate (Figure 8b), the texture is similar to the texture observed for sample manufactured at a low cooling rate (Figure 8a). In the polar figure (220), the prevalent directions <111> and <010> are observed. A quite different situation is observed for the sample manufactured using a high cooling rate.

The Fe_3_Al alloy produced under these conditions does not show a specific texture, which is reflected in the fairly uniform distribution of the normalized density intensity. Only a very weak texture with a <100> type component is observed. Both in the micro- and macrotexture studies, the presence of privileged crystallographic orientations have been observed. In both cases, the privileged directions are [111], [001], and [110], and their equivalent directions are distinguished as well.

A strong <100> fiber texture that is parallel to the build direction has been reported in many papers and is observed for various alloys [28,29,30,31,32,33,34]. The formation of the texture shown in Figure 8 originates from the fact that in bulk sections, the grains grow upward through many layers, which favors the development of a coarse grain structure that has a texture optimized with the average growth conditions. After several layers, the optimum grain growth direction is the build direction because the beam is scanned backwards and forwards and rotated by 90° for each alternating layer [28,32]. The formation of the strong <100> fiber texture is a compromised result of grain growth. Since a grain grows in any one layer with an <100> direction normal to the inclined melt pool surface, it will be tilted away from the build direction by an angle α. When the beam direction is reversed in the next layer, it will be aligned further away from the maximum growth direction by 2α, and thus is less favorably oriented. However, the grains with orientations that are closer to the maximum temperature gradient at the trailing edge of the melt pool may have an advantage for the growth in individual beam passes and will be poorly aligned when the beam travel direction reverses or rotates. Therefore, over many layers, the grains that are aligned with the <100> direction parallel to the build direction will on average be more closely aligned to the maximum temperature gradient when the raster pattern is alternated and will dominate the texture [28,32]. It must be noted that the results of the macrotexture tests are consistent with the results of the microtexture measurements and the grain morphology observations.

## 4. Conclusions

In this work, the grain size and morphology, high- and low-angle boundaries fractions and micro- and macrotextures of the Fe_3_Al alloy fabricated by the LENS technique were discussed with respect to the sample cooling rate during the process. The cooling rate range was closely related to the technological parameters, enabling obtaining a material free from metallurgical defects, such as non-melted powder particles, porosity, cracks, etc. Due to the applied process window, the resulting range of cooling rate variation was relatively small (0.64 × 10^4^ K/s–2.6 × 10^4^ K/s). The cuboid samples, with height up to 5 mm, produced from binary Fe_3_Al alloy using the LENS technique, are characterized by a columnar grain structure. The grain morphology of the deposited material is mainly characterized by the presence of large columnar grains spread over several layers along the building direction, regardless of the applied cooling rate. Additionally, fine equiaxed grains near the substrate or previous layer surface are also frequently observed. The appearance of fine equiaxed grains is most likely associated with a local increase in the cooling rate. Moreover, the increase in the cooling rate results in the decrease in columnar grain size and the decrease in the fraction of the low-angle boundaries. Regardless of the changes in the cooling rate, the <100> macro- and microtexture are dominant in the Fe_3_Al alloy produced by the LENS technique. Moreover, the level of dispersion from the dominant <100> texture increases noticeably with an increase in the cooling rate. Finally, the results of the macrotexture tests are consistent with the results of the microtexture study and the grain morphology observations.

## Figures and Tables

**Table 1:** Manufacturing parameters of LENS samples and obtained sample cooling rates.

Laser Power	Feed Rate	Powder Feed Rate	Layer Thickness	Cooling Rate (K/s)		
(W)	(mm/s)	(g/min)	(mm)	Bottom	Middle	Top
200	5	1.5	0.2	0.7 × 10^4^	0.6 × 10^4^	0.6 × 10^4^
MPS ^1^	15	3.7	0.2	2.1 × 10^4^	1.6 × 10^4^	1.6 × 10^4^
300	20	6.5	0.35	2.3 × 10^4^	3.5 × 10^4^	2.6 × 10^4^

^1^ MPS—the melted pool size control in the loop of the laser power feedback.
